# Relationship Between Osteoarthritis and Postmenopausal Osteoporosis: An Analysis Based on the National Health and Nutrition Examination Survey

**DOI:** 10.7759/cureus.71734

**Published:** 2024-10-17

**Authors:** Zheyu Fang, Jiaxin Zhao, Yuan Zhang, Xin Hua, Jia Li, Xu Zhang

**Affiliations:** 1 Neurology, The First Affiliated Hospital of Wenzhou Medical University, Wenzhou, CHN; 2 Surgery, The Fourth Affiliated Hospital, School of Medicine, Zhejiang University, Yiwu, CHN

**Keywords:** nhanes, osteoarthritis, postmenopausal osteoporosis, stratified analyses, women

## Abstract

Introduction

Due to the increased prevalence of osteoarthritis (OA) and osteoporosis (OP) in women, urgent interventions are needed to reduce the risk of postmenopausal fractures in female OA patients. Moreover, the relationship between OA and OP remains contentious, emphasizing the need for further research to deepen our understanding. This study aimed to investigate the relationship between OA and postmenopausal OP using data from the National Health and Nutrition Examination Survey (NHANES).

Methods

We conducted a cross-sectional analysis using NHANES summary data from 2005 to 2010, 2013 to 2014, and 2017 to 2020. Multivariable logistic regression was employed to evaluate the association between OA and the risk of postmenopausal OP. Adjustments were made for potential confounding factors. Additionally, we conducted stratified analyses, which provided further insights into the association between OA and postmenopausal OP across various subgroups.

Results

The analysis revealed a significant correlation between OA and an increased risk of postmenopausal OP, with an odds ratio (OR) of 1.12 (95% confidence interval (CI): 1.07-1.17, P < 0.001) after adjusting for confounders. Stratified analyses revealed a significant association between OA and postmenopausal OP in obese and overweight individuals (OR 1.14, 95% CI 1.06-1.22, P < 0.001; OR 1.18, 95% CI 1.04-1.32, P = 0.008) and among former or current smokers (OR 1.20, 95% CI 1.08-1.33, P < 0.001).

Conclusions

The study underscores a significant association between OA and postmenopausal OP, particularly in obese, overweight, and smoking populations. Given the higher prevalence of OA and OP among women, understanding these associations could lead to improved strategies for reducing postmenopausal fracture risks. The study offers valuable insights and potential directions for future therapeutic approaches.

## Introduction

Osteoporosis (OP) is a systemic skeletal disease characterized by reduced bone mass and deterioration of bone microarchitecture [1]. Known risk factors include age, ethnicity, gender, body weight, and smoking [1,2]. OP can be categorized into primary and secondary types. Primary OP encompasses three main forms: postmenopausal OP (type I), which arises from estrogen deficiency following menopause; age-related OP (type II), predominantly occurring in the elderly due to the aging process; and idiopathic OP, for which the causes remain unclear [3]. Postmenopausal OP is a systemic condition marked by reduced bone mineral density (BMD), deterioration of microarchitecture, and decreased bone strength, resulting in an elevated risk of fragility fractures [4]. Following menopause, the decline in estrogen levels disrupts the balance of bone remodeling, leading to increased bone resorption. Estrogen not only promotes the differentiation and maturation of osteoblasts but also inhibits the formation of osteoclasts and induces their apoptosis, thus enhancing bone formation and reducing bone resorption [5,6]. Research has demonstrated a strong correlation between estrogen levels and BMD, highlighting its critical role in the prevention of osteoporotic fractures. It is estimated that one in two postmenopausal women will experience an osteoporotic fracture in their lifetime, which can result in significant pain, disability, and a considerable decline in quality of life [4,7].

Osteoarthritis (OA) is a prevalent degenerative joint disease primarily affecting one or multiple joints. It can manifest as degeneration in both small joints (such as those in the fingers) and large joints (like the knees and hips). Key features of OA include degradation of articular cartilage, damage to subchondral structures, proliferation of joint tissues, vascularization of the synovium, and instability of ligaments and tendons [8]. Typical symptoms comprise pain, morning stiffness of less than 30 minutes, and restricted joint mobility, often accompanied by crepitus or sounds during movement. In severe cases, OA can lead to joint instability and physical disability, significantly impacting the quality of life [9]. Individual risk factors include advancing age, female sex, mechanical stress, genetic predisposition, and obesity [9,10]. Due to contemporary lifestyles characterized by higher rates of obesity and increased average lifespan, its prevalence is continuously rising [11].

When OA is considered as an outcome, studies have found a significant correlation between postmenopausal women's lumbar spine T-scores and OA [12]. OP and OA are two major musculoskeletal health issues among elderly individuals, impairing daily functioning and increasing morbidity and mortality rates [2,13,14]. Due to women's heightened susceptibility to OA and OP [2,9-11,15,16], urgent interventions are needed to mitigate the risk of postmenopausal fractures in female OA patients. Moreover, further research is necessary to thoroughly understand the relationship between OA and postmenopausal OP.

## Materials and methods

Study design

The study conducted a cross-sectional analysis, considering OA as the exposure and postmenopausal OP as the outcome. We utilized the National Health and Nutrition Examination Survey (NHANES)​​​​​​​ dataset to perform an epidemiological analysis investigating the association between OA and postmenopausal OP.

Study population

NHANES is a comprehensive survey of the U.S. population that provides rich information on public health and nutrition. Data collected from the NHANES survey are publicly accessible, making it a valuable resource for researchers and data users. The methods and data collection procedures of NHANES are well-documented in previous publications and can be accessed on the NHANES official website (http://www.cdc.gov/nchs/nhanes.htm). The survey protocol undergoes ethical review and approval by the National Center for Health Statistics (NCHS) Research Ethics Review Board to ensure compliance with ethical standards. Written informed consent is obtained from all participants involved in the study.

**Figure 1:**
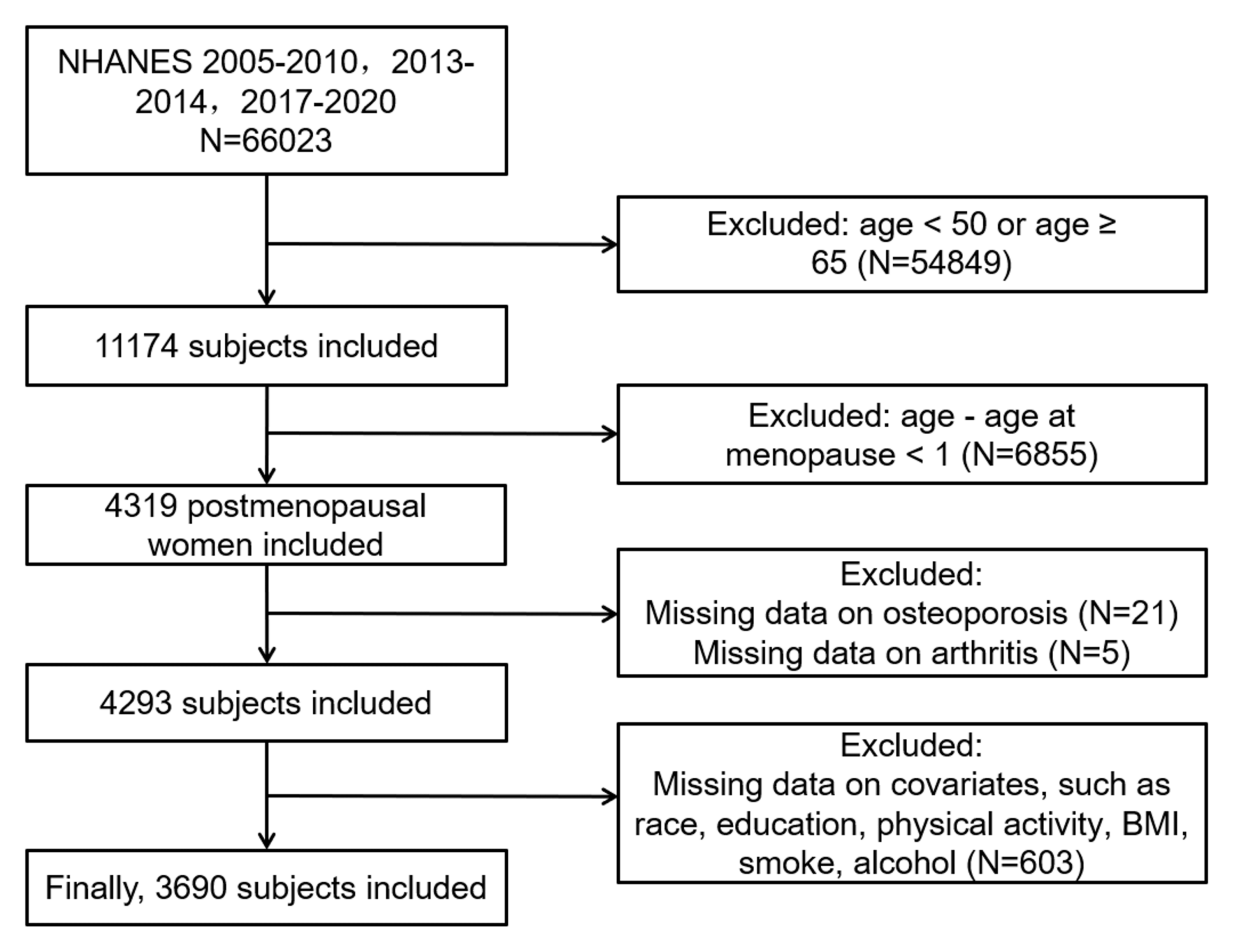
Screening procedure of study subjects NHANES: National Health and Nutrition Examination Survey

We conducted a cross-sectional study using NHANES data from 2005 to 2010, 2013 to 2014, and 2017 to 2020. During this period, a total of 66,023 individuals participated in the survey. Individuals under 50 or over 65 years of age were excluded, as well as participants who had been menopausal for less than one year. Additionally, individuals lacking relevant information (including OA, OP, race, education level, physical activity, BMI, smoking, drinking, and hypertension) were also excluded. Ultimately, 3,690 eligible participants were included, of whom 470 were diagnosed with postmenopausal OP​​​​​​​ (Figure 1).

Variables

The definition of OA was based on two main criteria: first, participants were informed by a physician or other health professional that they had arthritis; second, confirmation of the specific type of OA was obtained through specific questioning. Postmenopausal OP was defined as women aged 50 years and older but younger than 65 years, excluding OP due to advanced age, with their last menstrual period at least one year ago confirming entry into menopause, and having received a diagnosis of OP from a physician or other health professional. We included several categorical variables as covariates in the analysis: age, race/ethnicity, education level (high school or below vs. above high school), smoking status (non-smoker, former smoker, current smoker), excessive alcohol consumption, body mass index (BMI), hypertension, and moderate/excessive physical activity. Continuous variables such as age were also included in the analysis. Participants were asked about their lifetime smoking history, current smoking habits, and alcohol consumption, using two 24-hour recall methods for assessment. Former smokers were defined as individuals who had smoked at least 100 cigarettes in their lifetime but were not currently smoking. Excessive alcohol consumption was defined as an average daily intake exceeding 20 grams for men and 10 grams for women. If both 24-hour recalls were completed, alcohol intake was calculated as the average of the two recalls; otherwise, the data from the first 24-hour recall were used. BMI was categorized into four groups: underweight, normal weight, overweight, or obese (<18.5, 18.5-25, 25-29.9, ≥30 kg/m²). Vigorous/moderate physical activity was defined as participation in activities such as running or basketball or moderate-intensity activities such as brisk walking, cycling, swimming, or golf, lasting at least 10 minutes per session. Hypertension was defined as a systolic blood pressure greater than 130 mmHg or a diastolic blood pressure greater than 80 mmHg or the current use of antihypertensive medication.

Statistical methods

Our study followed NHANES sampling procedures and utilized complex survey weights to achieve national representativeness. Descriptive statistics summarized data as follows: normally distributed continuous variables are presented as mean ± standard deviation (SD), skewed variables as median (interquartile range (IQR)), and categorical variables as frequencies (%). The relationship between OA and postmenopausal OP was assessed using logistic regression models. Statistical analyses were conducted using R version 4.2.1 (The R Foundation for Statistical Computing, Vienna, Austria), with a significance set at P < 0.05.

Ethical statement

This study did not collect human tissue samples or clinical data. All data were obtained from publicly available online databases. The NHANES protocol was approved by the Institutional Review Board of the National Center for Health Statistics, and all participants provided written informed consent. The National Center for Health Statistics Ethics Review Committee granted ethics approval (protocols #2005-06, #2011-17, and #2018-01). Secondary analyses of these publicly accessible data were exempt from Institutional Review Board review.

## Results

Population-based study

The study enrolled a total of 3,690 participants. Table 1 summarizes the characteristics of female participants with postmenopausal OP. Among these participants, 470 individuals (13%) were diagnosed with postmenopausal OP. The mean age of the participants was 57.0 years (54.0, 61.0), with the majority being non-Hispanic White (1,385 cases, 72%). A total of 2,893 participants (87%) had completed high school education or higher. Additionally, 1,472 participants (47%) engaged in vigorous or moderate physical activity, and a significant portion was classified as obese (1,803 cases, 45%). Furthermore, 2,186 participants (56%) reported never having smoked, while 509 (18%) reported excessive alcohol consumption. Baseline results indicate significant differences between individuals with postmenopausal OP and those without in terms of age, BMI, OA, and smoking status. Postmenopausal osteoporotic patients may be more likely to have concurrent OA.

**Table 1:** Clinical characteristics of study participants Wilcoxon rank-sum test for complex survey samples; chi-squared test with Rao & Scott’s second-order correction *: statistically significant result (P < 0.05) BMI: body mass index; IQR: interquartile range Data are presented as median (IQR) for the continuous variable (age) and n (%) for categorical variables

Characteristic	Overall, N = 3690 (100%）	No postmenopausal osteoporosis, N = 3220 (87%）	postmenopausal osteoporosis, N = 470 (13%）	P-value
Age (median [IQR])	57.0 (54.0, 61.0)	57.0 (54.0, 61.0)	59.0 (56.0, 61.0)	<0.001*
Race (%)				0.2
Mexican American	566 (5.3%)	498 (5.5%)	68 (4.0%)	
Non-Hispanic Black	454 (4.6%)	382 (4.4%)	72 (5.7%)	
Non-Hispanic White	1,385 (72%)	1,191 (71%)	194 (75%)	
Other Hispanic	892 (11%)	802 (12%)	90 (7.9%)	
Other race - including multi-racial	393 (7.0%)	347 (7.0%)	46 (7.3%)	
Education level (%)				0.2
≤ High school	797 (13%)	690 (13%)	107 (17%)	
> High school	2,893 (87%)	2,530 (87%)	363 (83%)	
Vigorous/moderate physical activity (%)				0.8
Without	2218 (53%)	1924 (53%)	294 (52%)	
With	1,472 (47%)	1,296 (47%)	176 (48%)	
BMI (kg/m^2^）				0.016*
Underweight (<18.5)	55 (2.2%)	40 (1.9%)	15 (4.4%)	
Normal (18.5 to <25)	749 (25%)	637 (24%)	112 (33%)	
Overweight (25 to <30)	1,083 (27%)	944 (28%)	139 (25%)	
Obese (30 or greater)	1,803 (45%)	1,599 (46%)	204 (37%)	
Smoking (%)				0.002*
Current smoker	680 (18%)	567 (16%)	113 (28%)	
Former smoker	824 (26%)	720 (26%)	104 (23%)	
Never smoker	2,186 (56%)	1,933 (58%)	253 (49%)	
Excessive alcohol consumption (%)				0.8
Without	3181 (82%)	2767 (82%)	414 (83%)	
With	509 (18%)	453 (18%)	56 (17%)	
Osteoarthritis (%)				<0.001*
Without	3,104 (83%)	2,774 (85%)	330 (71%)	
With	586 (17%)	446 (15%)	140 (29%)	
Hypertension (%)				0.3
Without	2,819 (80%)	2,442 (80%)	377 (83%)	
With	871 (20%)	778 (20%)	93 (17%)	

Univariable and multivariable logistic regression analysis in postmenopausal OP patients

The results of the univariable logistic regression analysis assessing the risk of postmenopausal OP associated with OA are presented in Table 2, indicating a positive correlation (OR = 1.12, 95% CI: 1.07-1.17, P < 0.001). Additionally, age also exhibits a positive association with postmenopausal OP (OR = 1.01, 95% CI: 1.00-1.01, P < 0.001), while non-smoking status and obesity show a negative association. To elucidate whether OA independently impacts postmenopausal OP, a multivariable logistic regression adjusting for age, BMI, and smoking habits was performed (Table 2). After adjusting for these confounders, OA (OR = 1.12, 95% CI: 1.07-1.17, P < 0.001) remained significantly associated with the risk of postmenopausal OP. Taken together, OA may emerge as an independent risk factor for postmenopausal OP.

**Table 2:** Results of univariable and multivariable logistic regression analysis in postmenopausal osteoporosis patients Age is analyzed as a continuous variable, while the others are categorical variables *: statistically significant result (P < 0.05) BMI: body mass index; OR: odds ratio; CI: confidence interval

Variable	Univariable logistic regression analysis		Multivariable logistic regression analysis	
	OR (95% CI)	P-value	OR (95% CI)	P-value
Age (continuous)	1.01 (1.00, 1.01)	<0.001*	1.01 (1.00, 1.01)	<0.001*
Race				
Mexican American	Reference		Reference	
Non-Hispanic Black	0.99 (0.96, 1.03)	0.7	0.98 (0.94, 1.02)	0.3
Non-Hispanic White	1.04 (1.00, 1.08)	0.082	1.01 (0.97, 1.05)	0.7
Other Hispanic	1.07 (1.00, 1.14)	0.045*	1.06 (1.00, 1.13)	0.053
Other race - including multi-racial	1.04 (0.95, 1.13)	0.4	1.01 (0.93, 1.09)	0.9
Education level				
≤ High school	Reference			
> High school	0.96 (0.90, 1.02)	0.2		
Vigorous/moderate physical activity				
Without	Reference			
With	1.00 (0.97, 1.05)	0.8		
BMI (kg/m^2^)				
Normal (18.5 to <25)	Reference		Reference	
Obese (30 or greater)	0.94 (0.90, 0.98)	0.005*	0.93 (0.89, 0.97)	0.002*
Overweight (25 to <30)	0.95 (0.91, 1.00)	0.034*	0.95 (0.91, 0.99)	0.022*
Underweight (<18.5)	1.09 (0.87, 1.37)	0.4	1.10 (0.89, 1.36)	0.4
Smoking				
Current smoker	Reference		Reference	
Former smoker	0.91 (0.85, 0.98)	0.008*	0.92 (0.86, 0.98)	0.009*
Never smoker	0.91 (0.86, 0.96)	<0.001*	0.92 (0.88, 0.97)	0.001*
Excessive alcohol consumption				
Without	Reference			
With	0.99 (0.94, 1.05)	0.8		
Osteoarthritis				
Without	Reference		Reference	
With	1.12 (1.07, 1.17)	<0.001*	1.12 (1.07, 1.17)	<0.001*
Hypertension				
Without	Reference			
With	0.98 (0.94, 1.02)	0.3		

Stratified analysis of OA and postmenopausal OP incidence

**Figure 2:**
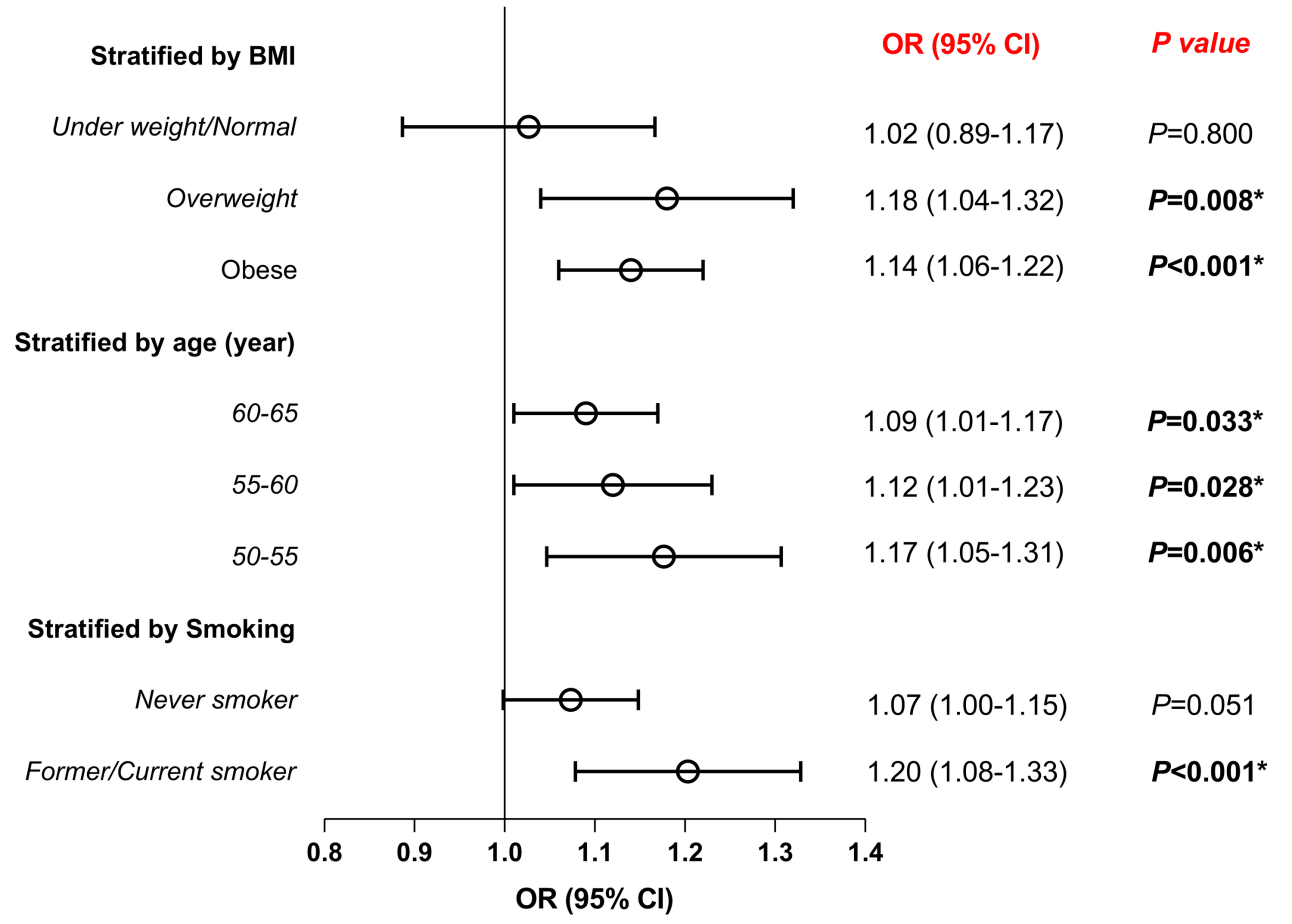
Associations between OA and postmenopausal osteoporosis based on BMI, age, and smoking status Logistic regression analysis *: statistically significant result (P < 0.05) BMI: body mass index; OR: odds ratio; CI: confidence interval; OA: osteoarthritis

Subsequently, we conducted stratified analyses, which revealed that the association between OA and postmenopausal OP remained consistent across all age groups. However, when stratified by BMI and smoking status, this association was not statistically significant in individuals who were under/normal weight or never smokers. OA showed a significant association with postmenopausal OP in obese and overweight individuals (OR 1.14, 95% CI 1.06-1.22, P < 0.001; OR 1.18, 95% CI 1.04-1.32, P = 0.008). Additionally, in former or current smokers, OA was also significantly associated with postmenopausal OP (OR 1.20, 95% CI 1.08-1.33, P < 0.001) (Figure 2).

## Discussion

This study employed large-scale observational research to investigate the relationship between OA and postmenopausal OP. In our cross-sectional study, we identified a significant correlation between OA and postmenopausal OP (OR = 1.12; 95% CI: 1.07-1.17, P < 0.001). Additionally, subgroup analyses indicated that OA was significantly associated with postmenopausal OP in obese or overweight individuals, as well as in patients with a history of smoking.

The relationship between OA and OP has been a topic of controversy [17]. A previous large systematic review found no significant association between overall OP and OA [18]. However, it is important to note that an increasing number of reports indicate a growing association between osteoporotic fractures and the progression of OA among affected patients. A retrospective cohort study, including 129, 348 OA patients and 129, 348 non-OA controls, confirmed a significant and positive association between OA and fractures across all genders, ages, and OA joint site subgroups [19]. Another retrospective cohort study compared elderly postmenopausal women with knee and hip OA to a control group without OA, revealing significant decreases in hip and spine BMD among those with OA [20]. Lower-limb OA patients are more susceptible to osteoporotic fractures [21]. Postmenopausal OP results from estrogen deficiency, which increases osteoclast differentiation and activation, accelerating bone resorption and exceeding bone formation [4]. Increasing evidence suggests estrogen plays a crucial role in maintaining joint tissue homeostasis. Studies in cells, animals, and humans provide compelling evidence that estrogen deficiency affects articular cartilage and other joint tissues in OA [15,16,22-23]. These findings further contribute to understanding the correlation between OA and postmenopausal OP.

Due to women's increased vulnerability to OA and OP, urgent measures are necessary to reduce the risk of postmenopausal fractures in women with OA [2,9-11,15,16]. To ascertain whether OA independently affects the risk of postmenopausal OP, we conducted a multivariable logistic regression analysis adjusting for age, BMI, and smoking habits. Even after accounting for these confounders, OA was significantly associated with postmenopausal OP. Stratified analyses further provided insights into the association between OA and postmenopausal OP across various subgroups. The association between OA and postmenopausal OP was consistent across all age groups, suggesting that age does not significantly modify this relationship. However, OA exhibited a significant association with postmenopausal OP in obese and overweight individuals. This suggests that excess body weight may exacerbate the risk of OP in individuals with OA. This is not contradictory to our conclusion regarding BMI as a protective factor from the univariable and multivariable logistic regression analyses. Specifically, while a higher BMI may be negatively related to the incidence of postmenopausal OP, the comorbidity risk between OA and postmenopausal OP remains significant in overweight and obese populations. Obesity is considered a risk factor for OA, as it increases the load on the joints, thereby promoting the development of OA [9]. The progression of OA may lead to joint pain and restricted mobility, which in turn reduces the patient’s level of physical activity. A lack of exercise may further exacerbate the risk of OP [24]. Therefore, while low body weight may be associated with the occurrence of OP, the impact of obesity on OA and its indirect effects, such as reduced physical activity, also play an important role in increasing the risk of OP. This indicates that although BMI may serve as a protective factor for postmenopausal OP in some contexts, the relationship between OA and postmenopausal OP in specific populations, such as overweight or obese individuals, still warrants attention. Similarly, the association was significant in former or current smokers. These findings imply that smoking status might also play a role in modulating the relationship between OA and postmenopausal OP.

In conclusion, our study highlights the importance of recognizing the association between OA and postmenopausal OP. The findings highlight the need for targeted preventive measures, particularly in populations who are obese or smokers, to address the compounded risk of postmenopausal OP in the context of OA.

Limitations

While this study offers valuable insights into the relationship between OA and postmenopausal OP, it is not without its limitations. First, the study's reliance on database analysis with datasets primarily drawn from European and American populations may limit the generalizability of the findings to international contexts with varying health conditions and healthcare systems. Therefore, further research is needed to validate these findings for broader application across different populations. Moreover, OA and postmenopausal OP share common risk factors, including age, gender, and BMI, which complicate the determination of a causal relationship between the two conditions. They may co-occur in patients with similar risk factors rather than being directly causally linked. When assessing Hill's criteria for temporality, while certain criteria such as the strength and consistency of the association may be met in the relationship between OA and postmenopausal OP, the cross-sectional nature of this study prevents the establishment of a clear causal link. Additionally, the diagnoses of OA and postmenopausal OP relied heavily on patient recall and self-reporting, without confirmation through objective medical examinations, potentially introducing bias. The study also did not explore the potential mechanisms that could explain the relationship between OA and postmenopausal OP, representing a significant gap in the research.

Despite these limitations, this study underscores the importance of developing effective management strategies to address these two interrelated diseases. Future research should aim to fill these gaps to better understand and manage these chronic conditions that affect a large patient population.

## Conclusions

In conclusion, this study highlights a significant association between OA and an increased risk of postmenopausal OP, emphasizing the need for targeted interventions in female OA patients. Utilizing data from the NHANES, our findings indicate that the odds of having postmenopausal OP are notably higher in individuals with OA, particularly among obese, overweight, and smoking populations. Based on the elevated prevalence of OA and OP among women, our results highlight the importance of early identification and management strategies to mitigate fracture risks. However, prospective cohort studies are essential to fully ascertain the relationship between OA and postmenopausal OP - whether it reflects a true causal relationship or merely a co-occurrence of two diseases in patients with similar risk factors. Future research should also investigate the underlying mechanisms linking OA and postmenopausal OP, as well as develop tailored therapeutic approaches to improve outcomes in affected populations.

## References

[1] Reid IR, Billington EO (2022). Drug therapy for osteoporosis in older adults. Lancet.

[2] Lane NE (2006). Epidemiology, etiology, and diagnosis of osteoporosis. Am J Obstet Gynecol.

[3] Zhang W, Gao R, Rong X, Zhu S, Cui Y, Liu H, Li M (2022). Immunoporosis: role of immune system in the pathophysiology of different types of osteoporosis. Front Endocrinol (Lausanne).

[4] Walker MD, Shane E (2023). Postmenopausal osteoporosis. N Engl J Med.

[5] Wu D, Cline-Smith A, Shashkova E, Perla A, Katyal A, Aurora R (2021). T-cell mediated inflammation in postmenopausal osteoporosis. Front Immunol.

[6] Fischer V, Haffner-Luntzer M (2022). Interaction between bone and immune cells: implications for postmenopausal osteoporosis. Semin Cell Dev Biol.

[7] Muñoz M, Robinson K, Shibli-Rahhal A (2020). Bone health and osteoporosis prevention and treatment. Clin Obstet Gynecol.

[8] Yokota S, Ishizu H, Miyazaki T, Takahashi D, Iwasaki N, Shimizu T (2024). Osteoporosis, osteoarthritis, and subchondral insufficiency fracture: recent insights. Biomedicines.

[9] Martel-Pelletier J, Barr AJ, Cicuttini FM (2016). Osteoarthritis. Nat Rev Dis Primers.

[10] Glyn-Jones S, Palmer AJR, Agricola R, Price AJ, Vincent TL, Weinans H, Carr AJ (2015). Osteoarthritis. Lancet.

[11] Molnar V, Matišić V, Kodvanj I (2021). Cytokines and chemokines involved in osteoarthritis pathogenesis. Int J Mol Sci.

[12] Tsai CJ, Wang YW, Chen JF, Chou CK, Huang CC, Chen YC (2023). Factors associated with osteoarthritis in menopausal women: a registry study of osteoporosis sarcopenia and osteoarthritis. J Family Med Prim Care.

[13] Fu Y, Kinter M, Hudson J (2016). Aging promotes sirtuin 3-dependent cartilage superoxide dismutase 2 acetylation and osteoarthritis. Arthritis Rheumatol.

[14] Katzir I, Adler M, Karin O, Mendelsohn-Cohen N, Mayo A, Alon U (2021). Senescent cells and the incidence of age-related diseases. Aging Cell.

[15] Pang H, Chen S, Klyne DM, Harrich D, Ding W, Yang S, Han FY (2023). Low back pain and osteoarthritis pain: a perspective of estrogen. Bone Res.

[16] Tang J, Liu T, Wen X, Zhou Z, Yan J, Gao J, Zuo J (2021). Estrogen-related receptors: novel potential regulators of osteoarthritis pathogenesis. Mol Med.

[17] Geusens PP, van den Bergh JP (2016). Osteoporosis and osteoarthritis: shared mechanisms and epidemiology. Curr Opin Rheumatol.

[18] Kim D, Pirshahid AA, Li Y, Varghese T, Pope JE (2022). Prevalence of osteoporosis in osteoarthritis: a systematic review and meta-analysis. Osteoporos Int.

[19] Jacob L, Kostev K (2021). Osteoarthritis and the incidence of fracture in the United Kingdom: a retrospective cohort study of 258,696 patients. Osteoarthritis Cartilage.

[20] Stamenkovic BN, Rancic NK, Bojanovic MR, Stojanovic SK, Zivkovic VG, Djordjevic DB, Stankovic AM (2022). Is osteoarthritis always associated with low bone mineral density in elderly patients?. Medicina (Kaunas).

[21] Anand V, Gupta A, Sethi S, Kumar S (2022). Study of relationship between bone mineral density in ipsilateral proximal femur and severity of osteoarthritis of knee. J Family Med Prim Care.

[22] Roman-Blas JA, Castañeda S, Largo R, Herrero-Beaumont G (2009). Osteoarthritis associated with estrogen deficiency. Arthritis Res Ther.

[23] Xiao YP, Tian FM, Dai MW, Wang WY, Shao LT, Zhang L (2016). Are estrogen-related drugs new alternatives for the management of osteoarthritis?. Arthritis Res Ther.

[24] Aibar-Almazán A, Voltes-Martínez A, Castellote-Caballero Y, Afanador-Restrepo DF, Carcelén-Fraile MD, López-Ruiz E (2022). Current status of the diagnosis and management of osteoporosis. Int J Mol Sci.

